# Poisoning with Arsenic—Late Discoveries

**Published:** 1840

**Authors:** 


### MECICAL JURISPRUDENCE AND TOXICOLOGY.

*On Poisoning with Arsenic:* ORFILA'S *Late Discoveries.*—The subject of poisoning by arsenic, which is so important both in forensic and practical medicine, has just been re-examined and thoroughly solved—at least in its medico-legal relations—in some memoirs communicated to the Royal Academy of Medicine.

For this we are indebted to M. Orfila. No one doubts the immense importance of the medico-legal applications of these new researches; but the same cannot be said of their therapeutic value, for this has been contested.

As we are disinterested witnesses of the discussion, and have conscientiously studied all the details of the question, we shall examine it successively under its two aspects. We must observe, however, that in these new inquiries of M. Orfila, the therapeutic question is subordinate to the medico-legal one. We shall, therefore, touch but slightly on the first; but we shall go at length into the method by which arsenic is detected *to a certainty*, in the bodies of those poisoned by it, and which, by the extension of which it is capable, will effect a fortunate revolution in toxicology, by ensuring the discovery of the greater number of poisons in the bodies of their victims; and this at every period of their crime, however advanced decomposition may have been, and in whatever manner the poison may have entered the living organs. Such, by anticipation, is the general enunciation of the results of these inquiries.

Hitherto, medico-legal examination in cases of poisoning has consisted chiefly in analysing the matters found in the stomach and intestines after death. Investigation was not pushed any farther.

But it often happens, however little the judicial inquiry may have been delayed, particularly when the dose of the poison has not been very large, that the most careful analysis does not detect the slightest trace of any poisonous substance, although poisoning has actually taken place. This is particularly the case with arsenic, the special object of the late investigations, and must be so also with a great number of other poisons.

The cause of the failure is obvious enough; the poison has been removed by absorption from the alimentary canal. Whether the arsenic is swallowed, or applied to any other part of the system, the explanation is the same; unless the dose is very large, it disappears sooner or later, being carried by the absorbents into the depths of the system. This being granted, it is plain that if we confine ourselves to testing for arsenic according to tha established methods, a skilful poisoner may escape tbe just punishment of his crime; for the jury is rarely satisfied except by the absolute detection of the arsenic.

Now, M. Orfila's method pursues the poison through every tissue of the frame, and detects it in the liver, the lungs, the brain, nay, in the last wrecks of organic matter. Here follows a proof ; we select it from many others, first, because it is decisive, and also because it has been the starting point from which all the datails of the question have proceeded.

On the 22d of December, 1838, a man died with all the symptoms of poisoning; but these are no more than probable evidence, as many natural diseases may be attended by the same symptoms. The body, therefore, was buried. A fortnight afterwards the public loudly demanded that the circumstances attending this death should be judicially investigated. The body was disinterred, and, on examining the digestive organs those anatomical lesions were discovered which usually follow poisoning by arsenic. It remained to be seen if chemical analysis would confirm the triple testimony borne by the symptoms of the disease, the organic lesions, and public opinion. Skilful experimenters tested and tested again, according to all the rules of science, but in vain : not an atom of arsenic was detected, and the corpse was again buried.

Meantime, the moral proofs of poisoning became stronger every day. M. Orfila was officially consulted, and, by his advice, the body was again disinterred, in April, about four months after it had been originally buried. The remains of the corpse were sent to Paris to be again examined. It is easy to imagine the state of the internal organs in a subject which had rapidly sunk under its symptoms, even if it did not perish by a violent death ; which had been buried and disinterred twice in the space of four months ; which had undergone all the minute trials of a judicial inquiry, as well at the hands of the chemists as of the physicians, and which, in conclusion, was exposed to the continual joltings of a post-chaise during a journey of eighty leagues. In fact, the stomach and intestines no longer bore any trace of organic structure, and all the other parts were more or less disfigured. It was to this mass of flesh, almost shapeless, and half decomposed, that M. Orfila had to apply his new method. This memorable experiment was performed in the presence of MM. Devergie and Lesueur, and with their assistance. The result did not disappoint the celebrated forensic physician ; he demonstrated the presence of arsenic in the liver and in the limbs of the victim ; and it was even detected in the cask which had been used to preserve the remains. Let us now mention in what this method consists, how it is practised, and on what its success depends.

Arsenious acid, when introduced into the stomach, or inclosed in the subcutaneous cellular tissues, is absorbed, and mixing with the blood, enters every organ. When it is finely powdered, and placed on the subcutaneous cellular tissue, only one or two grains are absorbed, whatever may be the quantity used; and this small quantity is sufficient to kill dogs of different sizes. More of it is absorbed when it is introduced into the digestive cavity.

The cases of poisoning which have hitherto occurred in man show that arsenic acts in the same manner that experiment shows it does upon dogs, except that a greater quantity is required to produce death in man. The mode of action of arsenic being thus determined, in reference to the parts to which it is applied, to the passages through which it makes its way, and the quantity which is required for poisoning, M. Orfila meets the questiou of the extraction of the poisonous substance, in his ordinary way, by experiments and facts. He first shows that it is possible to obtain metallic arsenic from the portion which has been absorbed; and next, that it is indispensable to have recourse to this extraction when the poison has not been found in the alimentary canal, nor in the matters vomited, nor in the other parts to which it may have been applied; for if we confine ourselves, as has been done hitherto, to looking for arsenic in the matters coming from the stomach and intestines, we run the risk of finding none, either because none remains in the digestive tube, or because the matters vomited have been removed; while the metal may always be obtained from the portion of the arsenious acid which has been absorbed, as facts and experiments continue to show.

This poison, adds M. Orfila, may be detected by properly treating a certain number of muscles, or a single viscus, previously dried, particularly if the viscus is very vascular; but it it is better to act upon the whole corpse, or at least on half of it, as the quantity of arsenious acid which has been fatal is too small for us to hope to detect it beyond the reach of doubt, if we treat only a single viscus, or an inconsiderable portion of the muscles and bones.

Moreover, arsenious acid can be detected in the blood obtained by bleeding the patient, provided there are several ounces to examine. In this point of view, at any rate, bleeding would be highly useful, by facilitating the inquiries of justice. We shall afterwards say what we think of its utility as a therapeutic agent.

We have just seen, on the one hand, how arsenious acid may be introduced into the system, and on the other, what becomes of it and by what line of proceeding it is to be detected; it remains only to show the method of extracting it. M. Orfila proves by the accumulated results of cases and experiments, that the best method of obtaining the metal consists in boiling the whole body in distilled water for six hours, in precipitating the impregnated fluid with sulphuric acid, then removing the arsenic from the sulphur which is deposited, mixing the decanted and filtered liquid with solid azotate of potass, (nitre,) evaporating the mixture to dryness, and then incinerating the product: this is first to be treated with water, and afterwards with concentrated sulphuric acid, and then to be put into Marsh's apparatus, not in its usual form—for this is inadequate to the purpose—but as modified M. Orfila. It would be disadvantageous to omit throwing down the precipitate with sulphuric acid, and to mix the liquid at first with the nitrate of potash, because whatever we do, we always lose a portion of the arsenious acid while burning the collected matter with the nitre. The loss will be evidently much smaller if we begin by removing from the fluid all that sulphuric acid can precipitate, and only treat with nitre the liquid which covers the precipitate.

It is proper to add that we lose but little arsenic if we burn the organic matter after having diligently mixed it with the dissolved nitre; while much less is obtained if the mixture of the animal matter and the nitre has been made in a morter. The loss is still more sensible, if incineration has been performed after Rapp's method.

The body is to be cut into pieces, and may be conveniently boilded in large cast-iron cauldrons, or in copper ones, if the verdigris is carefully removed; and a very clean iron pan, or a Hessian crucible, may be used for the decomposition of the animal matter by the nitre.

In places where, for want of utensils, the examiners do not undertake all these investigations, it will always be possible, and, indeed, is indispensable, to boil the body in a large cast-iron cauldron, for six hours, with distilled water and tea, or twelve grains of solid potash prepared by alcohol; and then evaporate the fluid to dryness, after having passed it through fine linen, while still luke-warm. The solid product may afterwards be conveniently submitted to the necessary tests. Lastly, the presence of arsenious acid in a human body with which it has not been placed in contact, provided its existence has been demonstrated by boiling the corpse cut into pieces, in distilled water, for six hours, without the addition of an acid, incontestibly proves that the poison has been taken during life; for numerous experiments have shown that the bodies of those who have not been poisoned with arsenic, when treated in the same manner, furnish no trace of it.

The chief object of the investigation which we have just analysed is to detect the presence of arsenic, either during the illness of the patiet, or after his death; and we are compelled to conclude that M. Orfila's new method of investigation, joined to his improvements of the old ones, makes it impossible henceforth to miss the tangible proof of arsenical poisoning; so that those who have committed such a crime can never again escape the vengeance of the law.

But there is another duty, of which the medical jurist must not lose sight; it consists in preventing the judges from committing an irreparable error by showing beyond all doubt, that the poisonous substance discovered in the living or dead body has been really introduced by a criminal hand, and that it can neither have been naturally produced in the human frame, nor artificially developed by the action or reaction of the numerous agents employed in testing.

M. Orfila has not overlooked the importance of this task. He first inquired whether the human body contained arsenic among its chemical principles; under what form it appeared; and whether it was possible to distinguish it from arsenic introduced from without. These points having been cleared up, he examined the conditions of the purity of the tests used, and fixed rules by which we may be assured that the arsenic obtained is neither derived from the reagent, nor the vessel, nor from the earth where the corpse may have been long deposited.

M. Orfila has ascertained the existence of an arsenical compound in the human body. This compound, which he believes to be arseniate of lime, is found in a small proportion in the bones, and perhaps in other tissues. This important fact might make one fear that the occurrence of a natural salt of arsenic would affect the results of our analysis, and thus condemn us to a lamentable state of doubt as to the perpetration of the crime. It is fortunate, however, that the experiments of our great medical jurists have cleared up this fearful dilemma; for they have proved that the natural arsenical compound is not soluble in boiling the distilled water kept neutral; while, on the contrary, poison introduced into tne system is dissolved and consequently disengaged by this menstruum. The reagents employed in these inquiries are sulphuric and azotic (nitric) acids, potash prepared by alcohol, azotate of potash, (nitre,) water, iron and zinc. M. Orfila shows that the sulphuric acid of commerce sometimes contains arsenic in the state of arsenious and of arsenical acid, which might lead to error; but he also teaches the means of purifying it. The same may be said of nitric acid, if it has not been distilled over nitrate of silver. The potash used in these examinations never contains any; the iron and zinc may contain some, but it is easy to test them beforehand, and free them from it.

The instruments used in these investigations are cast-iron boilers, porcelain capsules, Hessian crucibles, flasks and test tubes. The boilers will never yield any arsenic to the fluid that they contain, provided it is kept neutral by the addition of potash prepared with alcohol. The porcelain capsules, the flasks and the tubes never yield arsenic; but it is necessary to rinse them with an alkaline solution when they have contained arsenical substances.

Lastly, some earths contain arsenic, and may thus make medico-legal examinations more complicated. However it is easy to test them, and to distinguish the particles of arsenic which they afford from those furnished by the body.

To sum up: the important inquiries of which we have given a short abstract, authorize us to conclude that poisoning by arsenious acid will in future be recognized under all circumstances; and that it will be ascertained without the chance of being confounded either with the presence of arsenical salts naturally existing in the human body, or with the accidental occurrence of arsenical compounds in the tests or instruments used, or in the soil where the body has been buried. In other words, in the memoirs that we have just analyzed, poisoning by arsenious acid is examined under every aspect, and illustrated by such an apparatus of cases and experiments that the irresistible authority of the best demonstrated truths is stamped on the method of which a particular application is described.

To complete this analysis we will add a few words on the employment of venesection in the treatment of poisoning by arsenic. In the first place, is bleeding indicated? It is not long since antiphlogistics were thought to be universally indicated; while, at present, another extreme prevails, and they are rejected almost every where. This is so much the case, that after having formerly combated the absurdity of treating every disease by this method alone, we have now frequently to contend in favor of these powerful agents. Arsenical poisoning is a striking instance in point, yet discrimination is necessary in this as in every other case. If it is meant that in this kind of poisoning bleeding is indicated in every patient, in all circumstances, and in all the phases of its course, the answer is obvious; but no one, we believe, goes so far as this. On the other hand, if any one denys that bleeding is a means of lessening the consequences of this poison, and asserts that it necessarily hastens the catastrophe, and that we ought instead to use stimulants at every stage of the case, he commits a palpable error, which is every moment refuted by facts.

The length to which this article has extended does not allow us to enter into the details of the treatment, which too often is useless.

Before we discuss the different points of this important question, we shall wait till the new experiments projected by the committee of the Academy have thrown some light on the difficulties by which it is still surrounded.

*Gazette Medicale de Paris*, 17 *Aug*. 1839.

